# Evaluating Dermal Bioactivity of Metal Additive Manufacturing Powders Using Human *In Vitro* and *Ex Vivo* Skin Models

**DOI:** 10.1021/acs.chemrestox.6c00100

**Published:** 2026-05-03

**Authors:** Alexander Persson, Zuzanna Łyczyńska, Mariam Shahata, Oleksandr Kotlyar, Magnus Engwall, Eva Särndahl, Marcus Ehrström, Keira Melican, Inger Odnevall, Andi Alijagic

**Affiliations:** 1 Inflammatory Response and Infection Susceptibility Centre (iRiSC), Örebro University, SE-701 82 Örebro, Sweden; 2 Faculty of Medicine and Health, School of Medical Sciences, Örebro University, SE-701 82 Örebro, Sweden; 3 Poznan University of Medical Sciences, 61-701 Poznan, Poland; 4 Division of Dermatology and Venereology, Department of Medicine Solna, Karolinska Institutet, Sweden and ME Gastro/HUD/Reuma, Karolinska University Hospital, SE-171 77 Stockholm, Sweden; 5 Man-Technology-Environment Research Center (MTM), Örebro University, SE-701 82 Örebro, Sweden; 6 Centre for Applied Autonomous Sensor Systems (AASS), Robot Navigation & Perception Lab (RNP), Örebro University, SE-701 82 Örebro, Sweden; 7 Department of Chemistry, Division of Surface and Corrosion Science, KTH Royal Institute of Technology, SE-100 44 Stockholm, Sweden; 8 AIMES—Center for the Advancement of Integrated Medical and Engineering Sciences, Karolinska Institutet and KTH Royal Institute of Technology, SE-100 44 Stockholm, Sweden; 9 Department of Neuroscience, Karolinska Institutet, SE-171 77 Stockholm, Sweden; 10 Nordiska Kliniken, SE-111-51 Stockholm, Sweden

## Abstract

Metal additive manufacturing (AM) relies on alloy feedstock powders that may come into contact with the workers’ skin during handling, yet skin-relevant data on metal release and biological reactivity remain limited. Here, we assessed the cutaneous bioactivity of the fine particle fraction of four gas-atomized Fe-based AM powders (316L stainless steel, Fe-powder A, and tooling steels B and C). Powders were sieved to <10 μm and characterized by scanning electron microscopy and X-ray photoelectron spectroscopy before and after incubation in artificial sweat (ASW). Metal biodissolution was quantified in ASW and keratinocyte culture medium using atomic absorption spectrophotometry. Cellular responses were evaluated in HaCaT keratinocytes using Cell Painting-based phenomics and multiplex cytokine/chemokine profiling and in an *ex vivo* full-thickness human skin explant model, including superficial barrier disruption, IL-8/CXCL8 quantification, and histological assessment. ASW exposure induced marked shifts in the outermost surface composition across powders, indicating sweat-driven surface transformation. Biodissolution was low and medium-dependent, with Fe dominating the release in ASW, and with an overall metal release remaining limited in cell culture medium. In HaCaT cells, MCP-1/CCL2, IL-6, and IL-8/CXCL8 were quantifiable but showed no significant changes following powder exposure. Cell Painting revealed subtle, shared phenotypic signatures, primarily involving mitochondrial-associated features, without evidence of broad cellular stress. In the *ex vivo* skin model, AM powders did not increase IL-8/CXCL8 secretion, the particles remained localized to the skin surface without detectable penetration, and coexposure with *Staphylococcus epidermidis* did not enhance bacterial colonization or induce inflammation. To the best of our knowledge, this is the first study that applies a human skin explant model to evaluate dermal responses to metal AM powders. Overall, the tested AM powders showed low short-term cutaneous reactivity under skin-relevant conditions, providing human-relevant evidence to inform occupational risk assessment in AM environments.

## Graphical abstract

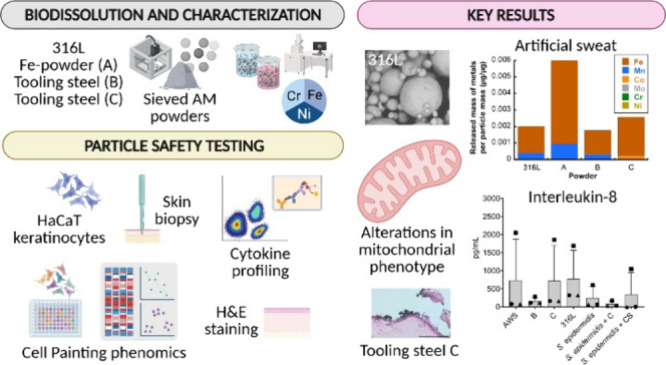

## 1 Introduction

Additive manufacturing (AM), commonly known as 3D printing, has rapidly expanded across industrial, medical, and research sectors.^1–4^ Among the various AM technologies, metal-based processes such as powder bed fusion and directed energy deposition rely on fine metal powders as feedstock materials.^5,6^ These powders often consist of alloys containing elements such as nickel (Ni), chromium (Cr), cobalt (Co), titanium (Ti), or aluminum (Al), and are handled during powder loading, part removal, postprocessing, and recycling steps.^7–10^ Biomonitoring studies from AM facilities have demonstrated measurable dermal contamination with metals such as Co and Ni, as well as corresponding increases in urinary metal levels among operators, indicating that dermal exposure represents a relevant occupational pathway.^11,12^ While respiratory risks of metal particles have received growing attention, considerably less is known whether they trigger local inflammatory or barrier-disruptive responses in human skin.^13,14^

The skin represents a primary barrier against environmental and occupational agents, but it is not an impermeable shield.^15,16^ Metal particles can deposit on the skin surface during handling and may interact with sweat, sebum, and the stratum corneum.^17–20^ Under these conditions, metal-containing particles may undergo partial dissolution, releasing metal ions that are, in some cases, known to contribute to skin sensitization, irritation, or inflammatory responses.^21–25^ The stratum corneum, the outermost layer of the skin, is often considered an effective barrier to metal absorption and can act as a reservoir for deposited substances.^26^ However, the available literature on the dermal absorption of metals remains limited. Several factors influence metal penetration, including pH, temperature, and the presence of salts, amino acids, proteins, and surface lipids on the skin.^26–29^ In particular, percutaneous penetration of metals is closely linked to their ability to form complexes or undergo oxidation in the presence of sweat.^30,31^ The extent of metal ion penetration is dependent on both skin integrity and exposure concentration, and absorbed ions may contribute to the development of allergic or inflammatory skin disorders over both short- and long-term exposure periods.^21^

Among metals, Cr, Co, and Ni, all present in AM metallic alloys, are well-recognized skin sensitizers and represent the most common causes of allergic contact dermatitis (ACD), and more rarely, allergic contact urticaria.^32^ Sensitization can occur even at low doses. It has been estimated that nickel allergy in the European general population remains high, estimated at approximately 8–19% in adults and 8–10% in children and adolescents, with a strong female predominance, while Co and Cr allergies are less prevalent, affecting around 1–3% of the population.^32–34^ Once these sensitizing metal ions penetrate the skin, they can trigger immune activation through multiple mechanisms. For example, Cr, Co, and Ni can interact with histidine residues in the human Toll-like receptor 4 (TLR4), activating pro-inflammatory signaling pathways and promoting the release of cytokines, such as tumor necrosis factor (TNF), interleukin-6 (IL-6), and interleukin-8 (IL-8/CXCL8).^35^ In addition, Ni and hexavalent Cr may stimulate cytotoxic T-cell responses through interactions with major histocompatibility complex (MHC) molecules.^35^

Despite this knowledge, the extent to which complex metal AM powders release bioavailable metal species under skin-relevant conditions and whether such release translates into measurable biological responses in human skin remain poorly characterized. This knowledge gap complicates risk assessment and the development of evidence-based safety guidelines for AM workplaces.

Traditional approaches for evaluating skin toxicity have relied heavily on animal testing or simplified *in vitro* assays that capture only limited aspects of skin biology. In recent years, there has been a strong push toward new approach methodologies (NAMs) that integrate advanced *in vitro* systems, high-content profiling, and human-relevant tissue models.^36,37^ These approaches allow for more mechanistic, sensitive, and ethically sustainable assessments of chemical and material bioactivity. In particular, high-content imaging methods such as Cell Painting-based phenomics enable unbiased phenotypic profiling of cellular stress responses,^38^ while cytokine measurements provide insight into inflammatory signaling. Complementing these cellular systems with particle biodissolution testing and *ex vivo* human skin models offers an opportunity to evaluate tissue-level responses in a structurally and biologically relevant context.

In this study, we applied an integrated testing strategy to assess the biological reactivity of metal AM powders in human skin models. We combined biodissolution testing in artificial sweat (ASW) and cell culture media with Cell Painting-based phenomic profiling and cytokine analysis in human keratinocyte (HaCaT) cells, alongside an *ex vivo* human skin biopsy model to examine the tissue level of inflammatory and histological changes. By linking material biodissolution behavior with cellular and tissue responses, this work provides a human-relevant assessment of the cutaneous bioactivity of different metal AM powders and contributes to the evidence base for occupational safety in AM environments.

## 2 Materials and Methods

### 2.1 AM Powders and Particle Size Separation

Four gas-atomized, Fe-based powders commonly used in AM were investigated. The materials were kindly provided by Swedish AM companies and included stainless steel (316L), one Fe-based powder (A), and two tooling steels (B and C). The nominal bulk compositions of the powders have been reported previously.^39^ To focus on the particle fraction most relevant for occupational exposure and biological interactions, the fine particle fraction (<10 μm) of the virgin powders was isolated, as smaller particles have a higher surface-area-to-mass ratio that may enhance interactions at the skin interface. Workplace measurements have demonstrated the presence of airborne AM-derived particles in the submicron and micron size ranges during handling and postprocessing tasks.^9^ A screenless air classifier (AC1000G, Blue Power, Germany) was used to separate particles <10 μm from the broader size distribution, as described earlier.^39^ Briefly, the method employs pressurized argon gas to pneumatically transport the powder through a cyclone equipped with a rotating classifier wheel. Smaller particles remain entrained in the gas flow, while larger particles are rejected and collected in a separate sampling vessel. The fine particle fraction is carried with the gas stream into a secondary cyclone, where it is collected. The classifier settings, including gas flow and wheel rotation speed, determine the particle size cutoff. Using this approach, mean cutoff sizes of <5 μm were achieved for powders A, B, and C, and <10 μm for the 316L powder.

### 2.2 Particle and Surface Characterization

Particle morphology and surface features of the size-classified powders were examined by using scanning electron microscopy (SEM; TM-1000, Hitachi, Japan). Imaging was performed in backscattered electron mode at an accelerating voltage of 15 kV. Powders were mounted on carbon adhesive tape to minimize particle dispersion within the chamber and to ensure adequate electrical conductivity during analysis.^39^

The surface chemical composition of the outermost particle layer (approximately 5–10 nm), before and after exposure to ASW, was analyzed by X-ray photoelectron spectroscopy (XPS) using an UltraDLD spectrometer (Kratos Analytical, UK). Powder samples were mounted on adhesive carbon tape for analysis. Measurements were conducted using a monochromatic Al Kα X-ray source (150 W), and spectra were collected from two separate surface areas (0.35 mm^2^ each) per sample. Both survey and high-resolution spectra were acquired (pass energy 20 eV) for the principal elements of interest, including Cr 2p, Ni 2p, Fe 2p, Mn 2p, Si 2p, Mo 3d, Co 2p, and the O 1s. All spectra were charge-corrected by referencing the C 1s adventitious carbon peak to 285.0 eV. The relative mass fraction of oxidized metals within the surface oxide layer was calculated and reported as the mean value from the two analyzed areas for each powder.

### 2.3 In Vitro Biodissolution Study

*In vitro* biodissolution experiments were conducted to assess metal release from the powders under skin-relevant conditions. Powders were suspended at a particle loading of 0.1 g/L in ASW (pH 6.5), following the EN1811 standard on test reference method for the release of Ni from products intended to come into direct and prolonged contact with the skin.^40^ Its chemical composition simulates the ionic composition and acidity of human perspiration. For each powder, three replicate samples and one blank control (ASW without particles) were prepared and incubated in parallel. All samples were exposed for 4 h in the dark at 37 ± 1 °C using an incubator with gentle bidirectional agitation (Edmund Bühler GmbH TH30, Bodelshausen, Germany). Agitation was applied at a maximum tilt angle of 12° and 22 cycles per minute to limit particle sedimentation and excessive agglomeration while maintaining the suspension. Following exposure, 10 mL of the supernatant was carefully collected and filtered through 0.02 μm syringe membrane filters to remove the suspended particles. The filtrates were acidified with 65% HNO_3_ to pH < 2 to stabilize dissolved metals prior to analysis. The concentrations of released metals were quantified using graphite furnace atomic absorption spectrophotometry (GF-AAS; PerkinElmer analyst 800) Detailed analytical procedures are described by Wang et al.^22^ The limits of detection (LOD) and quantification (LOQ) in ASW were 0.2 and 1 μg/L for Cr, 1 and 3.5 μg/L for Fe and Ni, and 1 and 3 μg/L for Co, Mn and Mo. Metal release data are presented as the mean ± standard deviation of three independent replicates per powder after subtraction of the corresponding blank mass values.

### 2.4 Cell Culture: Seeding, Exposure, and Sample Collection

Although not a primary inflammatory cell, HaCaT pose a relevant model for skin exposures and are capable of mounting a modest proinflammatory cytokine response.^41^ The human keratinocyte cell line HaCaT (Cell Lines Service GmbH, Germany) was maintained in Dulbecco’s modified Eagle medium (DMEM; Gibco) supplemented with 10% fetal bovine serum (FBS; Gibco), 4 mM l-glutamine (Gibco), 1 μg/mL gentamicin (Gibco), and 0.15 mM calcium. Cells were cultured at 37 °C under a humidified atmosphere containing 5% CO_2_. At approximately 70–80% confluency, cells were detached using TrypLE (Gibco), counted, and reseeded in fresh culture flasks for routine maintenance.

For experiments, HaCaT cells were seeded at a density of 15,000 cells per well in 96-well black-walled, clear-bottom PhenoPlates (Revvity, Waltham, MA) in a final volume of 100 μL of complete medium. After 24 h of incubation to allow cell attachment, cultures were exposed to the sieved metal powders for an additional 24 h in the total volume of 100 μL. Particle stock dispersions were freshly prepared for each experiment at a concentration of 10 mg/mL in Milli-Q water (18.2 MΩ·cm resistivity) and diluted in culture medium to a final nominal exposure concentration of 10 and 100 μg/mL. Two exposure concentrations (10 and 100 μg/mL) were used in the Cell Painting experiments and multiplex cytokine profiling, where 100 μg/mL represents an upper-range concentration commonly applied in *in vitro* particle screening,^12,39,42^ and 10 μg/mL was included to assess potential concentration-dependent effects. The selected concentrations are intended to represent a conservative *in vitro* exposure scenario, allowing the assessment of potential biological effects following dermal particle deposition under occupationally relevant conditions. Due to the comparative experimental design, negative but not positive controls were included. Outer microplate wells were filled with PBS to reduce edge effect, and exposures were randomized to reduce plate-position effect. Following the 24 h exposure period, 50 μL of cell culture supernatant was collected from each well for cytokine analysis (see section below). Supernatants were centrifuged at 5000*g* for 5 min to remove residual particles and cellular debris, aliquoted, and stored at −80 °C until analysis. The remaining adherent cells were subsequently processed for Cell Painting-based phenomics as described below.

### 2.5 Multiplex Immunoassay

Cytokine and chemokine concentrations were quantified using the bead-based multiplex assay LEGENDplex Human Inflammation Panel 1 (BioLegend, San Diego, CA) according to the manufacturer’s instructions. Samples were analyzed on a Gallios flow cytometer (Beckman Coulter, Brea, CA). Cytokine/chemokine concentrations were calculated from standard curves using the LEGENDplex data analysis software.

### 2.6 Cell Painting-Based Phenomics

Following particle exposure, a slightly modified Cell Painting assay was performed as previously described.^43,44^ Briefly, the remaining culture medium was removed, and 30 μL of MitoTracker solution (Invitrogen; Thermo Fisher Scientific, Eugene, OR) diluted in prewarmed culture medium (DMEM/F12 + GlutaMAX; Gibco) was added to each well. Cells were incubated for 30 min at 37 °C. Subsequently, 10 μL of 16% paraformaldehyde (PFA) was added directly to each well, and the cells were fixed for 20 min at room temperature. After fixation, the staining solution was removed, and cells were washed twice with 70 μL of phosphate-buffered saline (PBS). A staining cocktail was prepared in 1× PBS containing 0.1% bovine serum albumin (BSA) and 0.1% Triton X-100, supplemented with the following dyes: Hoechst 33342 (Thermo Scientific) for nuclei, SYTO 14 Green (Invitrogen) for nucleoli and cytoplasmic RNA, Concanavalin A/Alexa Fluor 488 (Invitrogen) for the endoplasmic reticulum, Wheat Germ Agglutinin/Alexa Fluor 555 (Invitrogen) for the Golgi apparatus and plasma membrane, and Phalloidin/Alexa Fluor 568 (Invitrogen) for F-actin. Cells were stained for 30 min in a final volume of 30 μL per well.

High-content images were acquired using an IN Cell Analyzer 2200 high-throughput imaging system (GE Healthcare, Uppsala, Sweden). Morphological features were extracted using CellProfiler (v4.2.1; https://www.cellprofiler.org) with established illumination correction (JUMP_illum_LoadData_v1.cppipe) and analysis (JUMP_analysis_v3.cppipe) pipelines (https://github.com/broadinstitute/imaging-platform-pipelines/tree/master/JUMP_production). Subsequent data processing included quality control, normalization to negative control wells (cells exposed to medium only), and feature selection as described previously.^43,45^ Custom Python scripts were used for the downstream processing of cell profiles.

### 2.7 Human Skin Tissue

Healthy human skin was obtained as surplus from elective plastic surgery approved by the Regional Ethics Review Board in Stockholm (Regionala etikprövningsnämnd i Stockholm; approval numbers 2015/432–31, 2023–00567–02, and 2024–06663–02). Written informed consent was obtained from all of the donors.

### 2.8 Ex Vivo Human Skin Culture and Bacterial Colonization

The *ex vivo* human skin explant model was prepared and maintained as previously described by Schulz et al. and Lang et al.^46,47^ Briefly, full-thickness 10 mm skin biopsies were generated using a biopsy punch (Agnthos, Stockholm, Sweden) and placed in 12-well transwell inserts (Sarstedt, Nümbrecht, Germany). Explants were supported with a CO_2_-independent medium (Gibco, Thermo Fisher Scientific; Cat. No. 18045088) supplemented with 10% fetal bovine serum (FBS) and 1% GlutaMax (Thermo Fisher Scientific). A superficial epidermal scratch was introduced to the explants using a 26G syringe needle tip (B, Franklin lakes, NJ). Particles were suspended in ASW at a final concentration of 100 mg/mL. To minimize sedimentation, the dispersion was vortexed frequently throughout the procedure. For bacterial conditions, *Staphylococcus epidermidis* 1457 ^48^ was grown in TSB at 37 °C (Tryptic soy broth, Sigma-Aldrich; Cat. No. 22092) to the log phase and then washed in PBS (Thermo Scientific; Cat. No. 10010023). 2 × 10^5^ colony-forming units (CFU) of *Staphylococcus epidermidis* 1457 was added onto the skin along with the metal particles (2 μL from the stock dispersion). Materials were applied to the center of each skin biopsy and allowed to air-dry before explants were returned to the incubator and maintained at 37 °C. After 24 h of incubation, skin biopsies were homogenized in 500 μL of PBS with a 1× HALT protease inhibitor (Thermo Scientific, Thermo Fisher Scientific; Cat. No. 87785) using FastPrep Lysing Matrix A tubes. Homogenization was performed at 6 m/s for three cycles of 20 s in a FastPrep-24 homogenizer (MP Biomedicals, San Diego, CA). For bacterial determination, serial dilutions were plated on tryptic soy agar (TSA) and incubated at 37 °C to quantify CFUs. The remaining homogenates were centrifuged at 16,000*g*, and the collected supernatants were stored at −20 °C for subsequent analyses.

### 2.9 Cytokine Detection

Interleukin-8 (IL-8) concentrations were quantified using the IL-8/CXCL8 DuoSet ELISA kit (Cat. No. DY208; R&D Systems, Minneapolis, MN) in 96-well half a rea Costar assay plates (Corning, Merck, Darmstadt, Germany), following the manufacturer’s instructions, but with half volumes, as previously described in Lang et al.^47^ 50 μL of capture antibody, sample, and detection antibody were used. Samples were not diluted. Absorbance was measured at 450 nm with a wavelength correction at 570 nm using a BioTek Synergy Mx microplate reader. Optical density values were analyzed using Microsoft Excel (version 16.79.1) and GraphPad Prism (version 10, GraphPad software Inc., La Jolla, CA).

### 2.10 Hematoxylin and Eosin (H&E) Staining of Skin Biopsies

Hematoxylin and eosin staining was performed as previously described in Lang et al.^47^ In brief, skin biopsies were fixed overnight in 4% paraformaldehyde, embedded in OCT, and cryosectioned at 12 μm. Sections were stained with hematoxylin (15–30 s) and counterstained with erythrosin B (5 s). Slides were differentiated under running tap water and then dehydrated in graded ethanol. Slides were cleared in xylene and mounted. Images were acquired using a Zeiss Axioplan light microscope with a 20× objective (Zeiss, Oberkochen, Germany).

### 2.11 Statistical Analysis

Statistical differences were assessed using one-way ANOVA, followed by Tukey’s post hoc test for multiple group comparisons. For Cell Painting data, unpaired Student’s *t* tests were used to compare each exposure condition individually against the control. The significance was rated as *p* < 0.05. The statistical analysis was performed in GraphPad Prism (v. 10.4.; GraphPad software Inc., La Jolla, CA, USA) and online matrix visualization and analysis platform MORPHEUS (https://software.broadinstitute.org/morpheus/).

## 3 Results and Discussion

To evaluate the skin interaction potential of sieved metal AM powders, an integrated experimental strategy was applied that combined material characterization with human-relevant biological models (Figure 1). The study linked particle physicochemical properties and biodissolution behavior to cellular and tissue-level responses using an *in vitro* keratinocyte model, high-throughput phenotypic profiling, cytokine measurements, and an *ex vivo* human skin explant model. This multitiered approach enabled assessment of both subtle cellular stress signatures and more overt inflammatory and histological changes; together providing a comprehensive view of the cutaneous biological reactivity of the tested metal AM powders.

**Figure 1:**
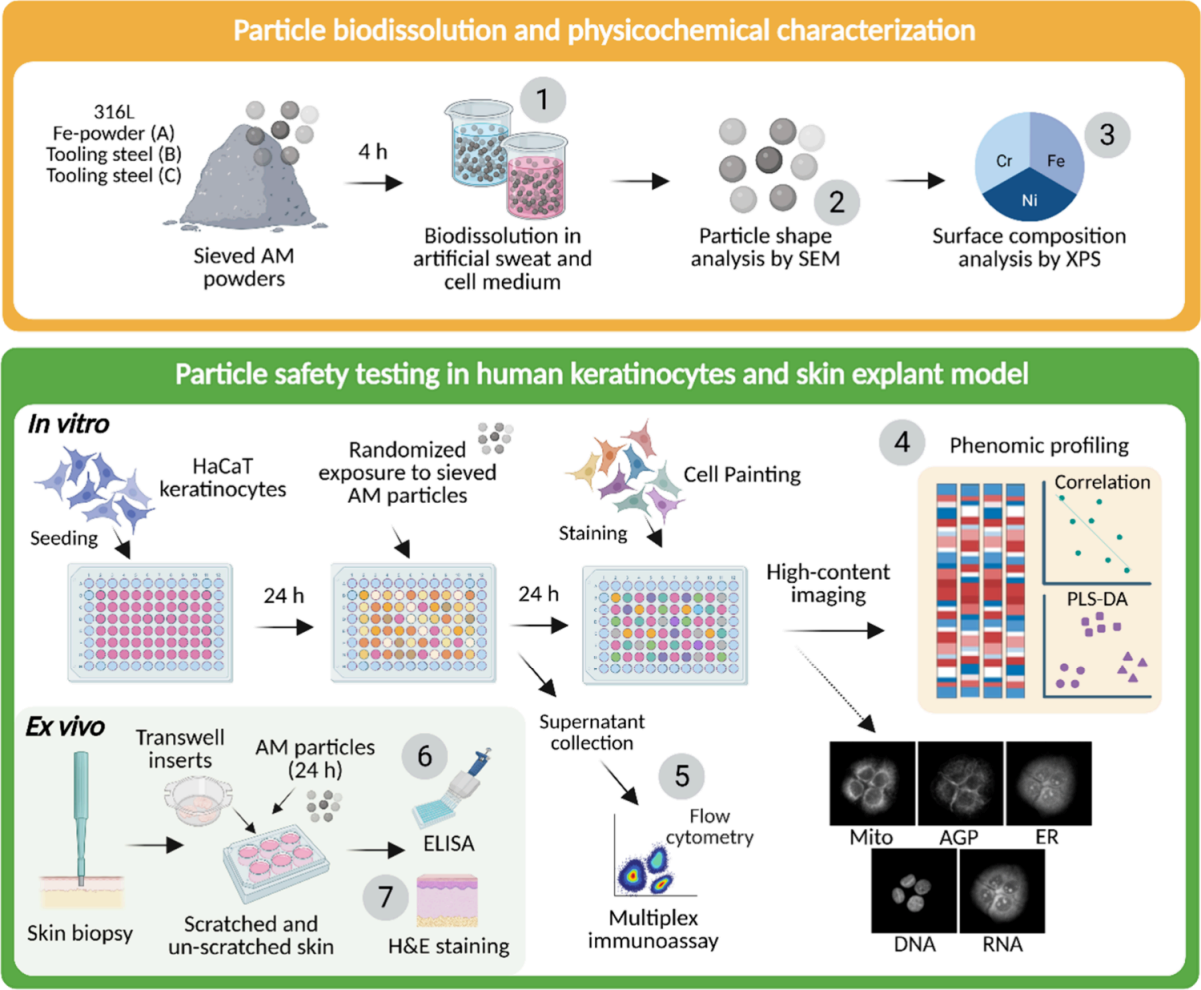
Study design and experimental workflow. Overview of the integrated approach used to evaluate the skin safety of sieved metal additive manufacturing (AM) powders. (1) Biodissolution testing of powders in artificial sweat and cell culture medium. (2) Particle morphology characterization was by scanning electron microscopy (SEM). (3) Surface chemical analysis by X-ray photoelectron spectroscopy (XPS). Biological assessment included both *in vitro* and *ex vivo* models: (4) cell Painting-based phenomics in human HaCaT keratinocytes, (5) cytokine and chemokine analysis in keratinocyte supernatants by multiplex flow cytometry, and *ex vivo* human skin explant experiments, including (6) interleukin-8 (IL-8) quantification by ELISA, and (7) histological evaluation using hematoxylin and eosin (H&E) staining.

### 3.1 Changes in Surface Chemistry of Sieved Metal AM Powders Following Exposure to Artificial Sweat

Particle shape and surface morphology of the sieved AM powders (316L, Fe-powder A, tooling steel alloys B and C) were examined by SEM (Figure 2A–D). Most particles were smaller than 5 μm, with a minor proportion approaching approximately 10 μm in size, consistent with previous characterization of these materials.^39^ A small number of particles exceeding 10 μm was also observed in the 316L sample (Figure 2A).

**Figure 2:**
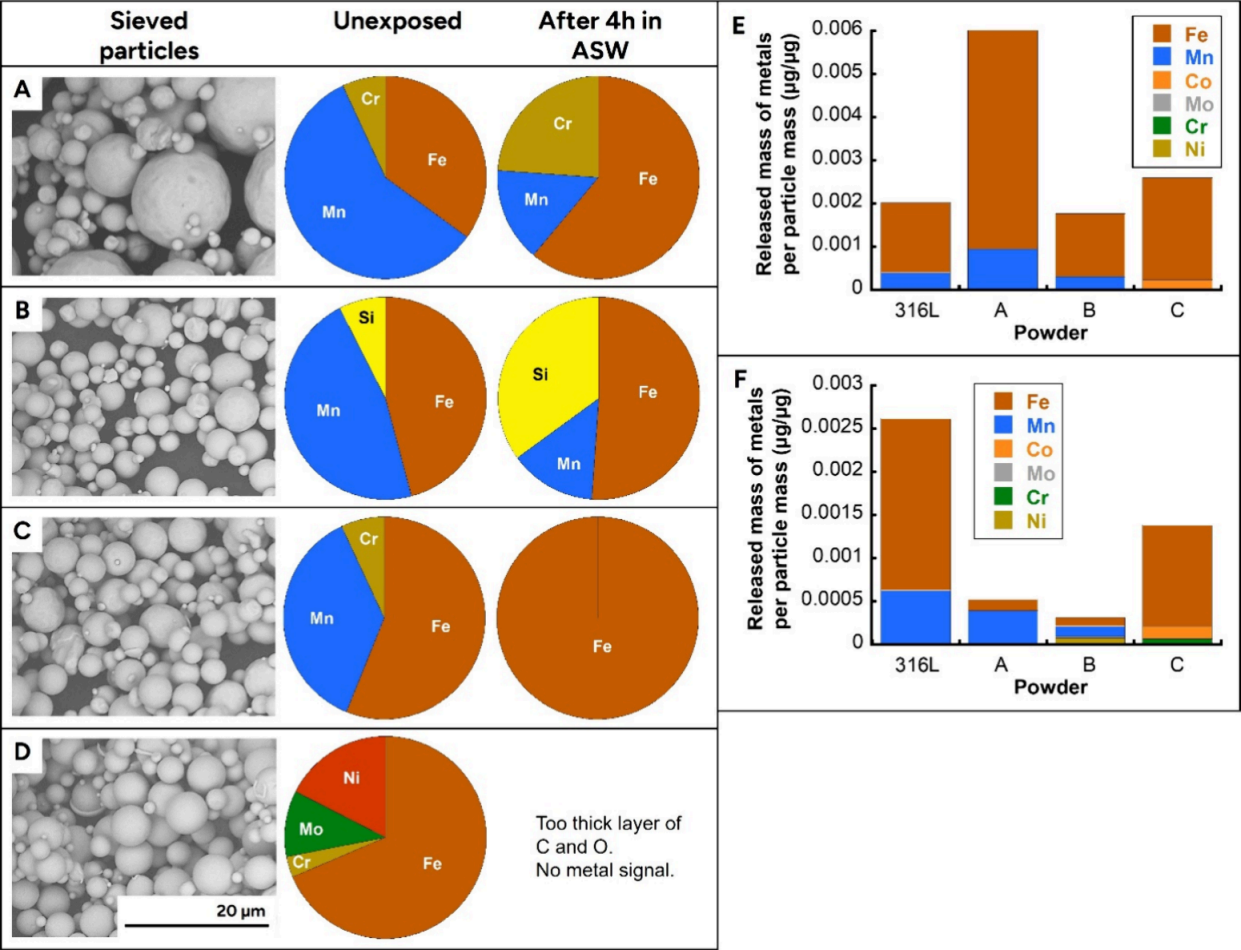
Particle morphology, surface chemistry, and metal release of sieved additive manufacturing (AM) powders. Scanning electron microscopy (SEM) images and X-ray photoelectron spectroscopy (XPS) of outermost surface composition of sieved particles before and after 4 h immersion in artificial sweat (ASW) for (A) 316L, (B) Fe-powder A, (C) tooling steel alloy B, and (D) tooling steel alloy C. Metal release normalized to particle mass after 4 h exposure is shown for (E) ASW and (F) cell culture medium (DMEM).

XPS analysis revealed differences in the elemental composition of the outermost surface layer among the powders prior to exposure to ASW. The surfaces of 316L, Fe-powder A, and tooling steel alloy B showed a high relative abundance of manganese, exceeding 50% of the detected surface metal fraction in the first two powders (Figure 2A,B). Tooling steel alloy C was characterized by the presence of Ni (Figure 2D), while Cr was detected in addition to Fe in the outermost top (5–10 nm) surfaces of 316L, tooling steel alloy B, and tooling steel alloy C (Figure 2A,C,D). In addition, tooling steel alloy C exhibited molybdenum (Mo) in the outermost surface layer, whereas Fe-powder A showed the presence of silicon (Si).

After 4 h of incubation in ASW, notable changes in surface composition were observed (Figure 2A–D). For 316L, the relative abundance of oxidized manganese (Mn) decreased markedly, while oxidized Fe and Cr became more prominent (Figure 2A). In Fe-powder A, oxidized Mn was reduced, and oxidized Si became strongly enriched, accounting for approximately 30% of the relative surface composition (Figure 2B). The surface of tooling steel alloy B became dominated by oxidized Fe following ASW exposure (Figure 2C). In contrast, for tooling steel alloy C, a thick surface layer rich in carbon and oxygen was detected after exposure, likely representing an organic or oxidized overlayer that attenuated the metal signal in the XPS analysis (Figure 2D). These surface modifications are highly relevant, as the outermost particle layer represents the primary interface with biological systems, including cell membranes and receptors. Understanding how this surface evolves under skin-relevant conditions such as exposure to ASW is therefore essential for interpreting subsequent biological responses.

### 3.2 Metal Release from Sieved AM Powders Is Low and Medium-Dependent

To evaluate the potential bioavailability of metals under skin-relevant conditions, biodissolution of the sieved metal AM powders was quantified in ASW and HaCaT keratinocyte cell culture medium by means of AAS (GF-AAS/F-AAS). Metal release profiles differed between the two media (Figure 2E,F). In ASW, Fe was the most abundantly released metal for all four powders (Figure 2E). Mn release was observed from 316L, Fe-powder A, and tooling steel alloy B, while tooling steel alloy C released low amounts of Ni. Considering the total quantity of released metals, Fe-powder A showed the highest overall metal release after 4 h in ASW. In the cell culture medium, a different release pattern was observed (Figure 2F). The highest total metal release occurred from 316L, followed by tooling steel alloy C. As in ASW, Mn was released from 316L, Fe-powder A, and tooling steel alloy B. In addition, tooling steel alloy B released small amounts of Ni, while tooling steel alloy C released low levels of Co and Cr.

Overall, the amounts of metals released in both ASW and cell culture medium were substantially lower than those previously reported in artificial lysosomal fluid (ALF).^39^ This difference is expected as ALF represents a more acidic and chemically aggressive environment that promotes metal dissolution, whereas ASW and cell culture media better reflect extracellular exposure conditions relevant to skin contact. Although workplace studies have primarily focused on inhalation exposure, it is known that AM operators can encounter fine and nanoscale particles during powder handling, cleaning, and postprocessing tasks.^9,12^ Our findings extend this knowledge by demonstrating that, under skin-relevant conditions, such particles exhibit limited metal release.

### 3.3 AM-Sieved Powders Do Not Induce Release of Inflammatory Mediators in Human Keratinocytes

To evaluate whether exposure to sieved AM powders triggered inflammatory signaling in human skin cells, we analyzed the release of cytokines and chemokines from HaCaT keratinocytes after 24 h of exposure using a multiplex immunoassay. Of the 13 analytes included in the panel, three mediators, MCP-1/CCL2, IL-6, and IL-8/CXCL8, were present at quantifiable levels in the cell culture supernatants (Figure 3). Across all tested powders and concentrations, no significant increases or decreases were observed in the concentrations of these cytokines/chemokines compared with unexposed control cells. These findings indicate that 24 h exposure to the tested AM powders did not elicit a measurable proinflammatory response in human keratinocytes under the experimental conditions used.

**Figure 3:**
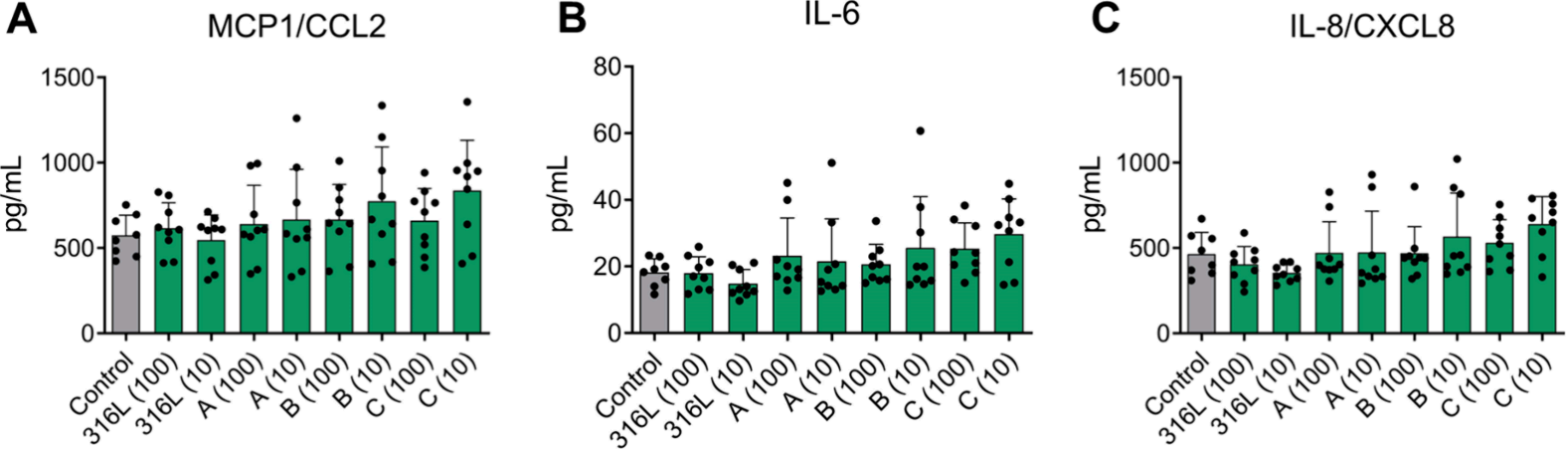
Cytokine and chemokine release from HaCaT cells exposed to sieved metal additive manufacturing (AM) powders. Cytokine and chemokine levels in supernatants from HaCaT cells exposed for 24 h to sieved metal powders at two concentrations (values 10 and 100 in parentheses on the *x*-axis indicate exposure concentration in μg/mL), including stainless steel (316L), Fe-powder A, and tooling steels B and C. Data are presented as mean ± standard deviation from three independent experiments (*N* = 3), each performed with three technical replicates. MCP-1/CCL-2, monocyte chemoattractant protein-1; IL-6, interleukin-6; IL-8/CXCL-8, interleukin-8.

The limited inflammatory response observed may be related to several factors. Given the relatively large particle size (<10 μm) and the applied concentrations (10 and 100 μg/mL), only a fraction of cells is likely to have come into direct contact with particles. In addition, metal release in cell culture medium was low (Figure 2F), suggesting the limited bioavailability of dissolved metal species capable of stimulating inflammatory pathways. Some variability between independent experiments was observed for certain powders, although control responses remained consistent. This variability likely reflects the inherent challenges associated with achieving identical particle deposition and dispersion across experiments when working with particulate materials.

These findings agree with our previous work using the same sieved AM powders in human macrophages, where the overall immune activation was limited. In that study, only minor increases in IL-8 were observed for powder A, and very modest elevations in MCP-1 were detected for powders A and B.^39^ Similarly, Vallabani et al. reported minimal inflammatory responses following exposure to AM-related particles, including spatter and condensate particles from a range of metal alloys such as IN939, Hastelloy X, 18Ni300 maraging steel, 316L stainless steel, and Ti6Al4 V.^42^ It is worth noting that the time frame of the exposures (24 h) reflects the acute inflammatory responses, and no conclusions can be drawn concerning potential effects of prolonged exposures of days and potential weeks.

However, not all previous studies are fully aligned with these observations, particularly regarding 316L. For example, Olšovská et al. investigated nanoparticles generated during selective laser melting of 316L in a human whole-blood model and reported measurable immune modulation, including a dose-dependent increase in TNF alongside suppression of IL-8 and only limited changes in IL-6.^49^ This pattern highlights that AM-derived nanoscale particles can influence acute inflammatory signaling in immune-cell–rich systems, even following the relatively short exposure time and when overt cytotoxicity is absent. In contrast, the present study focuses on keratinocytes, which are nonimmune cells and may require either higher bioavailable ion flux, stronger danger signals, or barrier compromise to produce robust cytokine outputs under short exposure windows. Likewise, Tang et al. showed that metal powders used for AM (15–100 μm; including 316L, CP-Ti and Ti-6Al-4 V) can elicit macrophage responses that depend strongly on particle concentration and size, with limited effects at low concentrations but more evident responses at high particle burdens; importantly, 316L tended to trigger a stronger proinflammatory cytokine profile than titanium-based powders.^50^ These differences were linked to surface chemistry (Fe/Cr oxide-rich surfaces for 316L vs Ti dioxide (TiO_2_) for Ti-based materials) and morphology features such as satellites/irregularities.

### 3.4 Cell Painting Phenomics Reveals Phenotypic Signatures of AM Powder Exposure in Human Keratinocytes

To capture potential early and subtle cellular effects of sieved AM powder exposure, a Cell Painting-based phenomics approach was applied as a broad and sensitive screening strategy. Unlike conventional cytotoxicity assays that primarily detect overt cell damage, Cell Painting enables high-content, single-cell-resolved profiling of phenotypic features across multiple cellular compartments. Changes in the cell phenotype are closely linked to alterations in cellular physiology, stress responses, and future functional behavior, making this method well suited to detect low-level bioactivity that may precede measurable toxicity.^51^ This unbiased, multiparametric approach therefore provided a sensitive tool to assess whether exposure to fine AM powders induced subtle phenotypic shifts in human keratinocytes before the onset of overt inflammatory or cytotoxic effects.

Despite the overall low impact on cell phenotypes, the heatmap summarizing all quantified features revealed a shared pattern across the tested metal AM powders (Figure 4A). The most responsive subcellular compartment for the four sieved powders tested appeared to be the mitochondria, which showed alterations across several exposure conditions (Figure 4A, highlighted region).

**Figure 4:**
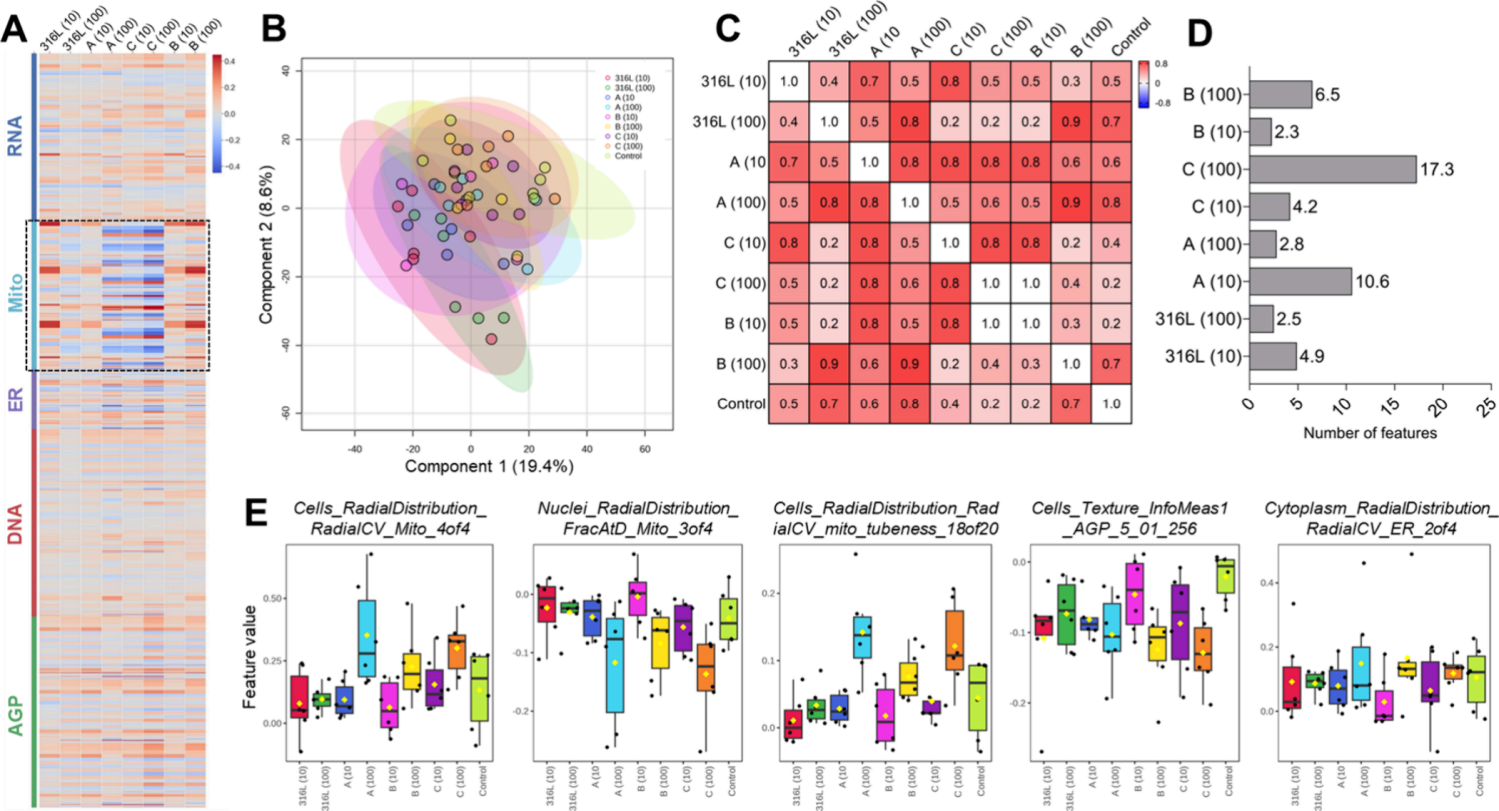
Cell Painting-based phenomics in HaCaT keratinocytes exposed sieved additive manufacturing (AM) powders. (A) Channel-clustered heatmap summarizing all quantified phenotypic features (total = 2,539), including the actin, Golgi, and plasma membrane (AGP) channel (642 features), DNA channel (630), endoplasmic reticulum (ER) channel (204), mitochondria (Mito) channel (497), and RNA channel (566). Colors indicate fold changes in feature values relative to the control cell profiles. Data are based on single-cell measurements aggregated across five technical replicates (wells) and six independent biological replicates (plates). (B) Partial least-squares discriminant analysis (PLS-DA) of all phenotypic profiles. (C) Correlation analysis showing Pearson correlation coefficients (*R*) between exposure conditions. (D) Percentage of significantly altered features (*p* < 0.05) per exposure condition relative to control keratinocytes, determined by *t* test. (E) Selected features from the AGP, Mito, and ER channels showing powder-specific phenotypic changes. Values of “10” and “100” in parentheses across all figure panels represent exposure concentrations (μg/mL).

Partial least-squares discriminant analysis (PLS-DA) demonstrated substantial overlap between control and exposed groups, indicating generally subtle effects; however, tooling steel alloy B at 10 μg/mL showed a tendency toward separation from controls (Figure 4B). Correlation analysis further supported the high similarity between phenotypic profiles, with some exposure groups (e.g., tooling steel alloy B at 10 μg/mL and tooling steel alloy C at 100 μg/mL) showing nearly identical profiles (Pearson’s *r* ≈ 1) (Figure 4C).

The proportion of significantly altered features (*p* < 0.05) was low across all exposure conditions, ranging from 2.3% for powder tooling steel alloy B at 10 μg/mL to a maximum of 17.3% for tooling steel alloy C at 100 μg/mL (Figure 4D). Selected features contributing to these changes were primarily associated with mitochondrial morphology and actin/Golgi/plasma membrane (AGP) texture (Figure 4E). This pattern is biologically plausible, as mitochondria are highly sensitive to subtle cellular stress and may respond even to low levels of metal exposure.^52^ Alterations in AGP texture are also consistent with the particulate nature of the exposures, as interactions between particles and the cell surface, potentially including adhesion or internalization, can influence cytoskeletal organization and membrane-associated structures.^53^ In contrast, features related to the radial distribution of the endoplasmic reticulum (ER) remained largely unchanged, suggesting that the ER organization and overall cellular architecture were not markedly disrupted under the tested conditions.

These data indicate that while AM powder exposure induced measurable phenotypic signatures in human keratinocytes, the effects were subtle and primarily reflected limited alterations in mitochondrial phenotype rather than widespread cellular stress or structural damage. Notably, these limited phenotypic shifts occurred in the absence of increased cytokine or chemokine release (Figure 3), supporting the conclusion that the detected responses reflect low-level cellular adaptation rather than overt inflammatory activation. This interpretation is further supported by the low metal release observed in cell culture medium (Figure 2F), indicating the limited bioavailability of soluble metal species capable of driving strong cellular responses. In addition, the ASW-induced surface transformations (Figure 2A–D) suggest that the outer particle surfaces under skin-relevant conditions may be dominated by relatively stable oxide or organic-rich layers, which could reduce the direct metal ion availability and reactivity at the cell interface. Collectively, the powder characterization and biodissolution findings provide a mechanistic context for the subtle phenotypic responses observed here, linking particle surface chemistry and limited metal release to the overall low biological reactivity of the tested AM powders in human keratinocytes.

### 3.5 AM Powder Exposure Does Not Induce IL-8 Secretion and Shows No Evidence of Penetration in Human Skin Explants

To examine the response of full-thickness human skin to AM metal powder exposure and corroborate the above-presented *in vitro* findings, a well-established *ex vivo* human skin explant model was employed. This model has previously been used to study inflammatory responses to bacterial colonization.^46,47^ In addition, a superficial scratch was introduced into the skin explants to mimic minor cuts and superficial wounds that commonly occur in occupational settings and may facilitate inflammatory responses. Application of the different AM metal powders at 100 μg/mL to *ex vivo* human skin explants did not significantly increased the IL-8 secretion compared with the negative control condition treated with ASW alone (Figure 5A). IL-8 levels measured after 24 h were comparable between metal powder-exposed and control samples, indicating that particle exposure did not elicit a detectable proinflammatory acute response under the tested conditions.

**Figure 5:**
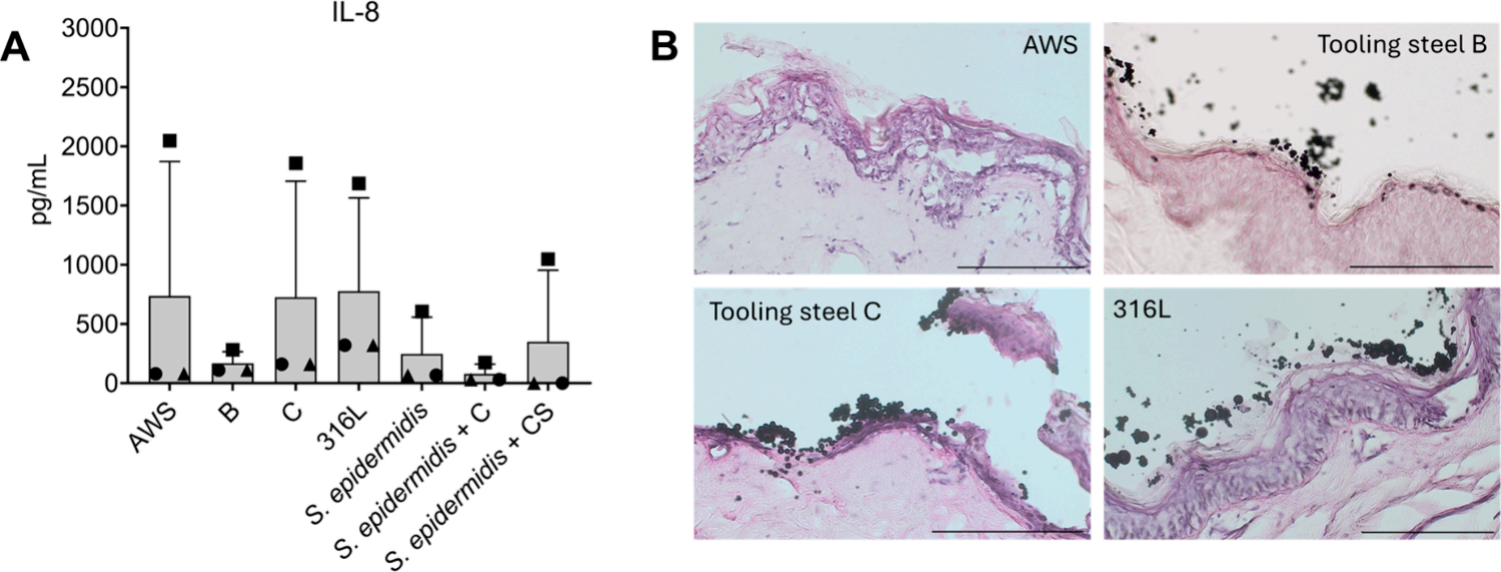
Inflammatory and histological responses of human skin explants to sieved additive manufacturing (AM) powders. (A) IL-8 secretion from *ex vivo* human skin explants after 24 h exposure to metal particles (100 μg/mL) suspended in artificial sweat (ASW). ASW served as a negative control. IL-8 concentrations were quantified by ELISA and are presented as the mean ± standard deviation, with individual donors indicated by different shapes. No significant differences were observed between particle-exposed and control conditions (*N* = 3 donors). IL-8 measurements were performed for 316L and tooling steel alloys B and C (Fe-powder A was not included in the *ex vivo* analysis). Labels “B” and “C” refer to different AM powders, while “CS” denotes coexposure conditions involving scratched skin. (B) Representative hematoxylin and eosin (H&E)-stained sections of human skin explants (*N* = 3) after 24 h exposure to selected AM powders (no bacterial coexposure). Scale bar = 100 μm.

In order to explore whether particle exposure influenced bacterial colonization or host responses to skin microbiota, explants were coexposed to particles and the commensal bacterium *Staphylococcus epidermidis*. No bacterial colonization (colony-forming units, CFU; data not shown) or increased inflammatory response (IL-8 secretion) was observed when *S. epidermidis* was applied together with particles compared with control conditions (Figure 5A).

Histological analysis further demonstrated that AM metal particles remained localized to the skin surface (Figure 5B). Microscopic examination showed that particles were confined to the outermost layers of the epidermis and did not penetrate deeper epidermal or dermal compartments. This surface localization persisted despite the mechanical disruption induced by superficial scratching prior to particle application, suggesting that minor barrier damage did not facilitate deeper particle penetration.

Together, these results demonstrate that under the experimental conditions used, the full-thickness human skin model did not mount an increased proinflammatory response to surface-deposited AM metal particles, even in the presence of common skin bacteria. The absence of IL-8 induction is consistent with the histological observations showing limited particle penetration. Notably, the introduction of superficial scratches, intended to simulate minor occupational skin injuries, did not enhance inflammatory responses or promote particle penetration.

These findings are consistent with occupational studies showing that AM operators experience dermal exposure to metals such as Co, Cr, and Ni, particularly during handling and postprocessing tasks, while systemic uptake remains generally low and influenced by work practices and protective measures.^11,54^ In addition, workplace studies have identified both direct skin contact with powders and surface contamination as relevant exposure pathways in AM environments.^9,12,54^ The present *ex vivo* results suggest that at least under 24 h exposure conditions and with only minor barrier disruption, these particles remain predominantly surface-associated and do not trigger measurable inflammatory responses. Taken together, the limited particle penetration and absence of cytokine induction support the interpretation that short-term dermal contact with these powders is unlikely to provoke acute inflammatory effects in intact or superficially disrupted skin. Further studies are needed to determine whether prolonged exposure or scenarios involving deeper barrier compromise could modify these outcomes.

## 4 Conclusions

This study provides a human-relevant assessment of the dermal bioactivity of the fine fraction (<10 μm) of four Fe-based metal AM powders (316L, Fe-powder A, tooling steels B and C) by integrating physicochemical characterization, biodissolution testing, and complementary *in vitro* and *ex vivo* skin models. Exposure to artificial sweat altered the outermost particle surface composition, indicating sweat-driven surface transformation, yet biodissolution remained low and strongly medium-dependent, with limited metal release in keratinocyte culture conditions. Consistent with this low bioavailability, HaCaT keratinocytes showed no significant induction of MCP-1/CCL2, IL-6, or IL-8/CXCL8 after 24 h of exposure, while phenomics revealed only subtle, shared phenotypic signatures dominated by mitochondrial-associated features and without evidence of broad cellular stress. Importantly, in a full-thickness human skin explant model, including superficial barrier disruption, AM powders did not increase IL-8 secretion, showed no detectable penetration beyond the skin surface, and did not enhance *S. epidermidis* colonization or inflammation.

Overall, the tested metal AM powders exhibited low cutaneous reactivity under skin-relevant 24 h exposure conditions. These findings support the use of integrated, human-based testing strategies to inform occupational risk assessment and guide evidence-based dermal safety practices in AM workplaces while highlighting the need for future studies addressing longer or repeated exposure scenarios to better reflect cumulative occupational exposure across workdays. Such conditions may reveal adaptive, low-grade, or delayed cellular effects not captured within a single 24 h exposure as well as the potential influence of skin damage or more reactive AM-derived particle fractions (e.g., ultrafine/nanoscale byproducts).
